# Relation of Crown Failure Load to Flexural Strength for Three Contemporary Dental Polymers

**DOI:** 10.3390/polym15214312

**Published:** 2023-11-03

**Authors:** Tariq F. Alghazzawi

**Affiliations:** 1 Department of Substitutive Dental Sciences, Taibah University, Madinah 42353, Saudi Arabia; tghazzawi@taibahu.edu.sa; 2 Department of Mechanical and Materials Engineering, The University of Alabama at Birmingham, Birmingham, AL 35294, USA

**Keywords:** polymer, flexural strength, composite resin, fiber, crown, bar

## Abstract

Polymeric materials show great promise for use in a variety of dental applications. Manufacturers generally provide flexural strength information based on standardized (ISO and ASTM) specimen dimensions and loading conditions. It is not clear, however, if flexural strength data are predictive of the clinical performance of dental crowns. The objectives of this study were, therefore, to determine whether flexural strengths, as measured via three-point bending (3PB), would be predictive of failure loads assessed via crunch-the-crown (CTC) tests. Three brands of polymers (Trilor, Juvora, and Pekkton) were fabricated into rectangular bars and fully contoured crowns (10 specimens of each polymer brand, 30 specimens of each shape). Differential scanning calorimetry (DSC), Fourier transform infrared spectroscopy (FTIR), and burn off tests were used to characterize/confirm the materials. Bars were tested blindly in 3PB to determine flexural strength, and crowns were CTC-tested to determine failure load after luting to resin abutments. The statistical significance of the test results was evaluated via one-way ANOVA (α = 0.05) and Pearson’s correlation coefficient, while regression analysis was used to test for a correlation between 3PB and CTC results. The fracture mechanisms and failure surface characteristics were characterized using scanning electron microscopy (SEM). There were significant differences (*p* < 0.05) in the mean crown failure loads (Trilor (7033 N) > Juvora (5217 N) > Pekkton (3023 N)) and mean flexural strengths of the bars (Trilor (468 MPa) > Juvora (197 MPa) = Pekkton (192 MPa)). The mode of crown fracture was different between the materials and included deformation (Juvora), ductile-to-brittle fracture (Pekkton), and a combination of cracks and deformation (Trilor). Flexural strengths did not correlate with the corresponding crown failure loads for any of the materials tested. These results suggest that dental practitioners should not rely on the flexural strengths reported from three-point bending tests, as advertised by the manufacturer, to predict the performance of polymeric crowns.

## 1. Introduction

Polymer-based materials are an ideal choice for dental applications that require bending, low density, and/or high adhesion [1,2]. A variety of monolithic materials are available, some of which can be reinforced with fibers or particles. These reinforcements generally increase the elastic modulus, strength, and wear resistance of the matrix [3]. The reinforcement of resin materials with fibers can increase mechanical properties and improve the clinical performance of dental restorations during their clinical function [4,5,6]. The improvement of composite resin properties depends on the fiber type (glass [7,8], polyethylene [7], or carbon [8]) and fiber orientation [7].

Polyaryletherketones (PAEKs) are a family of high-performance semicrystalline (crystalline and amorphous) materials. Two commercially available PAEKs for dental applications are polyetheretherketone (PEEK) and polyetherketoneketone (PEKK). PEEK is manufactured through computer-aided design and computer-aided manufacturing (CAD/CAM) technology, while PEKK may also be heat-pressed [9]. PEEK was initially applied to frameworks for fixed partial dentures [10,11,12] and removable partial dentures [13], implant abutments [14,15], surgical membranes [16], post cores [17], and implant bodies [18].

PEKK was introduced in dentistry more recently and has higher compressive strength and ductility and better long-term fatigue properties than unreinforced PEEK. PEKK has served as a framework for implant-supported, completely fixed dental prostheses because of its light weight [19]. Klur et al. [20] reported that PEKK can be used as a stable framework material for provisional fixed partial dentures; however, fractures of PEKK cantilever bridges were found to occur after a short time in service. Shams et al. [21] reported that PEKK coping veneered with cemented IPS e.max CAD can be an alternative to monolithic IPS e.max CAD endocrowns, in terms of fracture resistance. Rohr et al. [22] reported that Pekkton molar crowns on zirconia implants exhibited fracture load values similar to or higher than lithium disilicate crowns. Prechtel et al. [23] reported that 3D-printed and milled indirect PEEK molar inlays presented a higher fracture load than the expected physiological and maximum chewing forces. Mangoush et al. [24] reported that CAD/CAM-fabricated upper central incisor crowns made of single-structure short fiber-reinforced composites demonstrated encouraging performance related to their fracture behavior. Zimmermann et al. [25] reported a higher fracture load of particle-filled composite resin CAD/CAM crowns as compared to ceramic CAD/CAM crowns, for 0.5 mm thicknesses. The overall conclusion based on these efforts is that both PEKK and PEEK polymers show great promise for multiple dental applications.

Flexural strengths of dental biomaterials have been reported using three-point bending (3PB), four-point bending ($PB), and piston-on-ball (POB) tests. Rodrigues et al. [26] reported that flexural strength as measured via three-point bending was higher than that measured via four-point bending for microhybrids and nanofill composites. Miura et al. [27] reported that the biaxial flexural strength exceeded the three-point bending strength, which exceeded the four-point bending strength for dental hard resins. Pick et al. [28] reported that piston-on-ball (POB) tests better detected differences and displayed less data scattering for resin composites as compared with three-point bending tests. Additionally, results from POB tests were better at predicting failures of the material as estimated using finite element analysis. Winter et al. [29] reported that the fracture resistance and flexural strength of CAD/CAM polymer-based materials were lower than those of glass ceramics but still sufficient for use in the first molar region; however, they did not determine any correlations between the two mechanical properties. Rohr et al. [22] reported that a linear trend was found between the fracture load and the fracture toughness of Pekkton material.

Manufacturers generally provide flexural strength information to differentiate between materials based on standardized specimen dimensions and loading conditions, as dictated by the International Organization for Standardization (ISO) and American Society for Testing and Materials (ASTM) standards. It is not clear, however, if flexural strength data are predictive of the clinical performance of dental crowns, which motivated the present efforts. The objectives of this study were, therefore, to determine whether flexural strengths, as measured via three-point bending (3PB), would be predictive of failure loads assessed via crunch-the-crown (CTC) tests. It was hypothesized that the 3PB flexural strength would be predictive of CTC failure load for the three contemporary polymers. Differential scanning calorimetry (DSC), Fourier transform infrared spectroscopy (FTIR), and burn off tests were used to characterize/confirm material properties for three different polymer materials.

## 2. Materials and Methods

Three polymeric materials (Trilor, TRI; Juvora, JUV; Pekkton, PEK) were provided in a “blind” manner. The specifications of the test materials, including the identifier, manufacturer, class, Young’s modulus, and Poisson’s ratio, are shown in Table 1 [30]. The classification of mechanical properties according to the geometry and dimensions is detailed in Table 2. Test specimens were fabricated as follows: crown-shaped specimens were used to determine failure loads using CTC, and rectangular-shape bars were used to determine flexural strengths using 3PB. The sample size (*n* =10) was determined from past publications [10,12,31].

DSC was used to analyze the melting temperature and recrystallization temperature of the thermoplastic polymer matrix in the polymeric materials (SDT Q600, TA Instruments, New Castle, DE, USA). The heat flow was measured when the polymer absorbed or released heat during its melting or recrystallization, respectively. The DSC specimens were extracted from the edge of the polymer specimen which are small enough to fit into the DSC pan. A heat-cool-heat cycle in a N_2_ environment was carried out for the specimens. Specimens were heated at 10 °C/min to 400 °C, cooled at 10 °C/min to 20 °C, and then heated at 10 °C/min to 400 °C.

FTIR (Thermo Nicolet 4700 FT-IR optical spectrometer, Ramsey, MN, USA) was used to obtain the infrared spectra of the different polymeric materials. Each polymer has unique molecular bonds, which are displayed as signature peaks in the FTIR plot; therefore, this method can be used to differentiate polymers using databases and the literature [32,33]. The specimens were in sheet form and each specimen was clamped down during scanning. The 3730 scans were used for the specimens with a scan resolution of 1 cm^−1^.

Burn-off tests were conducted to measure the fiber weight percentage of the fiber-reinforced polymeric materials. The tests were carried out based on the procedure specified in ASTM D3171-2015 [34] to determine the constituent content of composite materials. The mass before burn off (initial mass M_i_) and after burn off (M_f_) was measured, and the following equation was used to calculate the fiber weight percentage W_r_:

W_r_ = (M_f_/M_i_) × 100. (1)

All crown and disc test specimens in the present study were fabricated in-house from raw discs, as provided by the manufacturer, and then machined from the same discs using advanced computer technology. The dimensions of the crown and bar specimens were, therefore, controlled, uniform, and standardized with very low standard deviations pertaining to their dimensions after fabrication.

To create consistent crown specimens for the CTC tests, an ivorine maxillary 1st molar tooth (Model #R861; Columbia Dentoform Corp, Long Island City, NY, USA) was prepared and then duplicated and poured with a resin material (Die Epoxy Type 8000, American Dental Supply, Inc. Allentown, PA, USA) to fabricate resin abutments (10 abutments per brand, height = 8 mm from the highest point of the occlusal surface to the base) [35]. The epoxy resin abutments were digitized using a 3D scanner (D2000, 3Shape A/S, Copenhagen, Denmark) in order to fabricate the crown shape specimens using a Wieland Mini milling machine (Wieland Dental + Technik GmbH & Co. KG, Pforzheim, Germany). The crown specimens were fabricated with the following specifications: cement gap = 0.075 mm, extra cement gap = 0.120 mm, and polymer thickness at the central fossa = 0.8 mm according to manufacturer recommendations.

The cementation technique followed that of previous publications [36,37], where Panavia V5 (Kuraray Medical Inc., Tokyo, Japan) was used to attach the crowns to the corresponding resin abutments. A load of 50 N was applied on the monobloc of the crown–resin abutment after the removal of excess cement [36,37]. All cemented crown-on-resin abutments were stored for 24 h in water at 37 °C.

A steel ball (diameter = 11.37 mm) was used in the CTC test to crush the cemented crown-on-abutment specimens using a mechanical testing machine (MTS 858 Mini-Bionix, MTS Systems, Eden Prairie, MN, USA) at a speed of 0.5 mm/min until failure [35]. A polyethylene sheet (0.9 mm thickness) was placed between the ball and the crown to distribute the load over the occlusal region [35]. The failure load was recorded as the maximum force for each test.

Rectangular bars were fabricated, and their edges were beveled using a Wieland Mini milling machine. The bar specimens were fabricated with the following dimensions: 2.0 ± 0.1 mm wide, 2.0 ± 0.1 mm thick, and 25.0 ± 2.0 mm long. A mechanical testing machine was used to load the bar specimens in a 3-point bending configuration at 0.5 mm/min until the specimens failed. The test span, l, was 20.0 mm. The flexural strength (MPa) was calculated according to ISO 20795-1:2013 standard [38]:

(2) $$\sigma =\frac{3Fl}{2bh^{2}}$$

where *F* was the maximum load in Newtons; *l* was the test span in mm; and *h* and *b* were the bar thickness and width, respectively, in mm.

Statistical differences between the experimentally determined mechanical properties were determined using one-way ANOVA with Tukey post hoc tests. Data transformation using rankings was used if needed to provide for equality of variances (Levene test) and to better normalize the distribution (Shapiro–Wilk test). The coefficient of Pearson’s correlation was used to determine the correlation between the flexural strength and failure load. Regression of mean values correlating various data points was performed using the data analysis tool in Excel. The results returned a correlation coefficient and *p* value.

Scanning electron micrographs (Quanta FEG 650 scanning electron microscope, FEI, Hillsboro, OR, USA) of the failed surface region for each specimen were obtained after experimental failure. These surfaces were first sputtered with gold palladium using a high-vacuum mode (accelerating voltage = 30 kV). Energy-dispersive spectroscopy (EDS) was conducted at different points to determine the polymer/fiber composition.

## 3. Results

**Figure 1:**
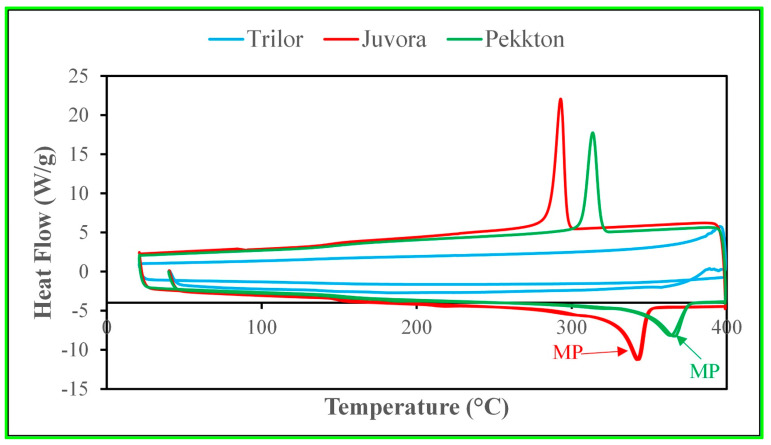
DSC of the materials where specimens were heated at 10 °C/min to 400 °C, cooled at 10 °C/min to 20 °C, and then heated at 10 °C/min to 400 °C. TRI: No melting peak is observed, indicating that it is a thermosetting polymer. JUV: A melting peak is observed, indicating that it is a thermoplastic polymer. The melting temperature is 342 °C, and the recrystallization temperature is 293 °C. PEK: A melting peak is observed, indicating that it is a thermoplastic polymer. The melting temperature is 366 °C, and the recrystallization temperature is 314 °C. DSC = differential scanning calorimetry, TRI = Trilor, JUV = Juvora, MP = melting point.

The DSC plots of heat flow versus temperature for different polymeric materials are shown in Figure 1. JUV and PEK revealed distinct melting peaks and recrystallization peaks, which confirmed that these two materials were thermoplastic polymers. However, no melting peaks or recrystallization peaks were noticeable in TRI, which is characteristic of a thermoset polymer matrix. These results confirmed the manufacturer specifications.

**Figure 2:**
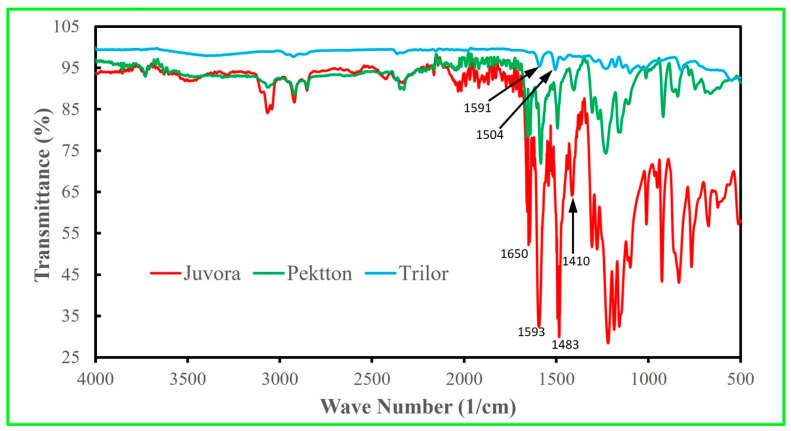
FTIR spectra (transmittance versus wave number) of JUV, PEK, and TRI. Comparison of the present peaks with the database indicated that JUV is PEEK, PEK is PEKK, and TRI has an epoxy matrix. Baseline correction and automatic smoothing were used. The spectrum of the JUV specimen (PEEK) shows carbonyl (C=O) stretching at 1650 cm^−1^ and skeletal ring (C=C) stretching at 1593 cm^−1^, 1483 cm^−1^, and 1410 cm^−1^. Similar peaks were found in the Pekkton (PEKK matrix) specimen at those wave numbers. The peaks of the TRI (epoxy matrix) sample at 1591 cm^−1^ and 1504 cm^−1^ show C=C stretching. FTIR = Fourier transform infrared spectroscopy, TRI = Trilor, JUV = Juvora, PEK = Pekkton.

**Figure 3:**
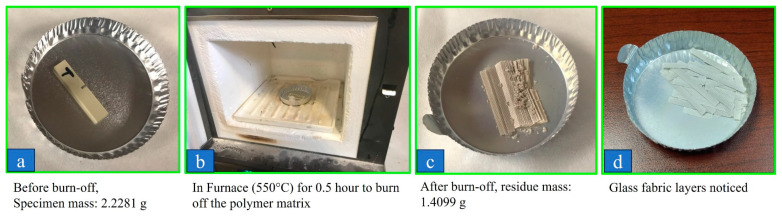
Burn off test results for TRI indicated that the weight percentage of the glass fibers in TRI was approximately 63 wt%. TRI = Trilor.

Figure 2 shows the FTIR plots of the intensity of the transmitted light against its wavelength. The comparison of the peaks with a database confirmed that JUV is PEEK and PEK is PEKK. The EDS for the surrounding region for the TRI had the elements of C and O, which could be the epoxy resin, which was further confirmed using the FTIR spectrum according to the database. The epoxy matrix of the TRI material was completely burned off in air as illustrated in Figure 3, and the residue was calculated to be 63 wt% woven glass fibers.

**Figure 4:**
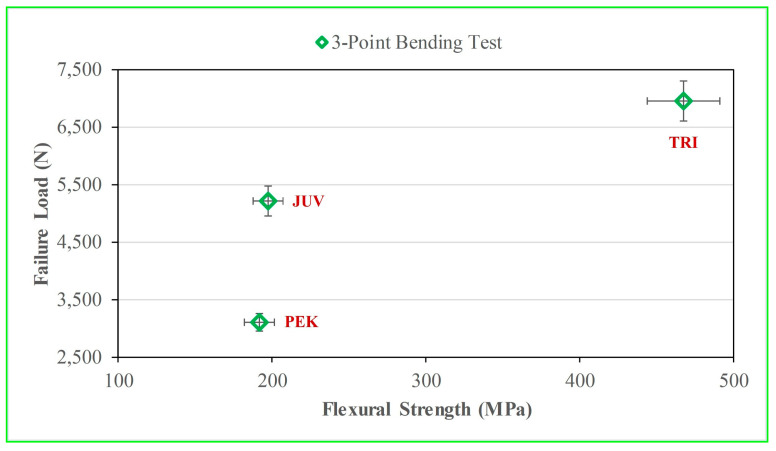
Flexural strength (bar specimens) with failure load (crown specimens). There was no correlation (R^2^ = 0.31 and *p* = 0.062 between flexural strength and failure load.

The mean and standard deviation for the flexural strength and failure load for each polymer are listed in Table 3. TRI had a higher flexural strength and failure load than PEK and JUV (*p* < 0.05). There was no significant difference (*p* > 0.05) in the flexural strength between JUV and PEK, as listed in Table 3. There was no correlation between flexural strength and failure load for any of the polymeric materials tested, as shown in Figure 4. The failure load (TRI > JUV > PEK) was not proportional to their corresponding flexural strength, as indicated by R^2^ = 0.31 and *p* = 0.062.

**Figure 5:**
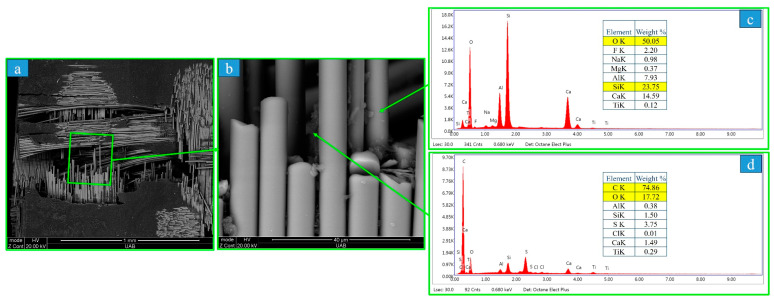
The BSE microphotographs of fractured surfaces after the three-point bending test for TRI at different magnifications ((**a**) = 100×, (**b**) = 2500×). The microstructure presented fibers with concentrations of Si and O, which confirmed that the fibers were glass (**c**) compared to the EDS for the resin matrix (**d**). BSE = backscattered (reflected) electrons, TRI = Trilor, Si = silicone, O = oxygen, EDS = energy-dispersive spectroscopy. C = carbon.

The SEM images illustrated in Figure 5 on the cracked surface of a TRI bar showed that the material comprised a woven fabric in a 0/90 weave pattern. The fiber diameter ranged from 12 to 14 µm. In addition, the fiber contained Si (24 wt%), Al (8 wt%), Ca (15 wt%), and O (50 wt%) elements (based on the EDS spectrum), as illustrated in Figure 5, which indicates that the fiber in TRI was glass, as expected. TRI specimens failed due to a combination of cracking and deformation at the occlusal surface of the crowns, as well as the center of the bars.

**Figure 6:**
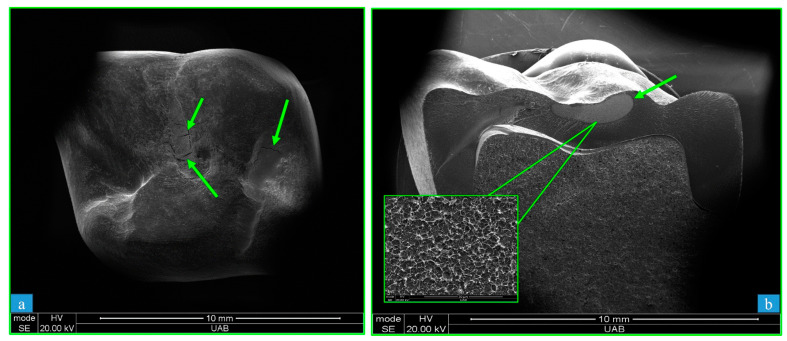
SEM micrographs of fracture surfaces of (**a**) = JUV (PEEK) at 20× and (**b**) = PEK (PEKK) at 21× crowns. The JUV crowns (**a**) failed due to crushing of the occlusal surface, with the remaining crown still intact. Surface cracking was observed on JUV irradiation. Dimple features characteristic of ductile fracture were evident for PEK crowns (**b**), which broke in half into two pieces as a result of occlusal surface pressure from the ball indenter of the CTC test. SEM = scanning electron micrographs, JUV = Juvora, PEK = Pekkton, CTC = crunch-the-crown. The arrows in “a” indicate cracks and in “b” indicate dimple features.

The mode of fracture was different for JUV and PEK materials (Figure 6). The JUV crowns (Figure 6a) failed due to surface cracking that did not progress, leaving the crowns relatively intact. In contrast, the PEK crowns (Figure 6b) fractured completely into two pieces, where the failure was characterized by ductile-to-brittle fracture, as shown by dimple formation (microvoid coalescence) underneath the occlusal surface, surrounded by brittle fracture surfaces. These images, along with additional SEM images of the TRI, JUV, and PEK failure surfaces, may be viewed in our previous manuscript [33].

## 4. Discussion

The three polymeric materials used in the present study were provided in a “blind” manner, so we did not know, a priori, which material was JUV, TRI, or PEK. The materials were, therefore, characterized using DCS and FTIR tests. DSC was performed to determine which polymeric material was thermoset (TRI) or thermoplastic (JUV and PEK) using melting and recrystallization peaks. The FTIR spectra were obtained to determine which material was PEEK (JUV), PEKK (PEK), and fiber-reinforced composite resin (TRI) using the database (the intensity of the transmitted light against the wavelength of the light) and further confirmed using spectra from other studies [34,39].

Presently, the composition of the three polymeric materials was confirmed with the use of EDS, by which JUV and PEK were found to have carbon and oxygen as their main elements (hydrogen cannot be captured via EDS). The TRI material presented fibers characterized in the SEM images, where the 12 to 14 µm diameters confirm that the fibers were woven glass (carbon fiber diameters are typically less than 10 µm), which was further confirmed with the burn off procedure. In the present study, the fibers were not completely oxidized, and there were residuals after burnout, which confirmed that the fibers were not carbon. Furthermore, the TRI displayed tooth coloring, which further confirmed that the fibers were glass, as carbon fibers produce dark specimens that are not suitable for dental applications.

In the present study, the flexural strengths obtained from the 3PB testing of bar specimens, created from three commercial dental polymers, were investigated for their correlation with failure loads obtained from the so-called crunch-the-crown (CTC) test. The goal of the study was to “bridge the gap” between a mechanical property determined from a controlled test and how the material may perform clinically. Flexural strength testing is a standardized approach with a defined size, shape, and loading conditions. The CTC test is intended to provide a reasonable indication of how a material will behave when it is fabricated into a crown, where the geometry is not simple and the stress state is more complex. In the present study, epoxy resin abutments were used instead of human molar teeth, because it would have been difficult to standardize the dimensions of the molars. Our use of resin abutments simulates human hydrated dentin (18 GPa) [40], since epoxy resin presents a similar elastic modulus (4 GPa) [41]. The 3PB test was used as recommended by ISO 20795-1 for testing the flexural strength of polymers [38].

The specimens were not polished in the present study because it is extremely difficult to standardize the polishing of the irregular occlusal surfaces of the crowns to the same consistency as bar specimens with standardized flattened surfaces and beveled edges. Thermocycling is not recommended by the ISO standard for testing flexural strength; therefore, the crowns were not exposed to any form of artificial aging (thermocycling), as this would have resulted in a different treatment from the bar specimens. We understand that the materials used in the present study are applied clinically as a core design and must be covered by composite resin for esthetic reasons [12]. We further recognize that the CTC tests do not fully simulate the clinical oral environment, where cyclic loads may be applied eccentrically as well as vertically and tensile stresses may be generated on the intaglio surface [10]. We do believe, however, that the CTC test represents a more clinically relevant test than a simple 3PB test, which was the motivation for the present study.

Presently, the measured failure loads for the crowns did not correlate with the flexural strengths found through the 3PB of corresponding bar specimens. Based on these findings, our hypothesis that the 3PB would be predictive of the CTC failure load of the different polymeric materials was rejected. These results were consistent with our previous findings for zirconia, where piston-on-three-ball (biaxial flexion) and four-point bending test results did not correlate with corresponding CTC test results [42]. In contrast, four-point flexural strengths were observed to positively correlate with CTC failure loads in glass ceramic materials; however, there was no correlation of CTC failure load with biaxial flexural strength [43].

Each of the three polymeric materials have been shown to resist the range of human biting forces [44], which makes them useful for dental applications. The fiber-reinforced TRI material may be more useful for frameworks of monolithic crowns and fixed partial dentures in posterior regions, however, because it showed higher failure load and flexural strength than JUV and PEK, which do not have such fiber reinforcement. The fibers are known to bear most of the externally applied load and contribute to most of the strength of composite materials as dictated by different fiber types, percentages, and orientations [7,8].

The failure loads for crowns composed of JUV and PEK were significantly different, and the mode of failure was different for each. The JUV specimens failed due to plastic deformation with cracks visible via SEM. The failed crowns and bars remained structurally intact. PEK specimens failed due to ductile-to-brittle fracture, whereby both the crowns and the bars were broken into two pieces. This result is due to the fact that JUV presents much higher ductility (up to 150% strain to failure), as compared to PEK (13% strain to failure).

The as-prepared TRI specimens presented exposed matrices and fibers after machining from the raw TRI discs. After loading the TRI specimens (both crowns and bars), cracks appeared, and they ultimately failed through a combination of plastic deformation and cracking. This ductile-brittle fracture mode likely resulted from cracking caused by matrix fracture, fiber fracture, and/or delamination between the glass fiber layers, the details of which are beyond the scope of this study. Any surface cracks exposing underlying fibers would have deleterious effects in the oral environment and may contribute to hygroscopic expansion [40]. Therefore, the differences in failure modes comparing the TRI and PEK specimens is likely attributed to the fibers that held the TRI specimens together.

In our study, the polymeric crowns were machined from solid discs with full contour design (0.8 mm) and cemented on resin abutments. They yielded fracture loads of 3023 ± 418 N for PEK and 5217 ± 169 N for JUV, which were higher than recorded maximum biting forces [43]. Rohr et al. [22] reported that the fracture load of milled molar PEK crowns on zirconia implants (ceramic implant, 4.0 mm) was 2921 ± 300 N. Shams et al. [21] reported that the fracture load for PEK crowns was 1831 ± 240 N. The reduction in failure loads compared to our study may have been a result of their veneering and thermocycling processes, as well as their endocrown designs. Elmougy et al. [40] reported that PEK crowns failed due to fracture with slight deformation at the occlusal surface at loads of 2037 ± 49 N, which is lower than the value obtained presently. This discrepancy is likely due to differences in crown thickness and the nature of the supporting resin material. Rodríguez et al. [10] reported that the fracture load for three-unit posterior fixed partial denture frameworks (0.7 mm occlusal thickness) with an intermediate pontic of PEEK material was 3132 ± 307 N. Prechtel et al. [23] reported that the mean fracture load for milled indirect PEEK inlays was 2981 N.

Elmougy et al. [40] reported a biaxial flexural strength of 227 MPa for Pekkton bars, whereas in the present study, the flexural strength from 3PB was 192 MPa. This discrepancy is likely a result of different loading conditions, as well as the fact that our specimens were not polished (as stated previously, we could not standardize the polishing procedures between the bars and crowns). Suzaki et al. [7] reported that the flexural strength as determined via 3PB was 254.2 ± 22.3 MPa for fiber-reinforced composite resin (TRINIA, SHOFU), as compared to 468 ± 97 N (TRI) in our study. The difference may be attributed to the fiber type and orientation. Shrivastava et al. [13] reported that the flexural strength of PEEK via 3PB was 183 MPa, which is close to 197 MPa in our study.

PEEK has shown promise as an alternative to titanium because the lateral stress on implants, as well as crestal bone loss, could be reduced in comparison with the titanium implants [45].

The results of this study suggest that dental practitioners should not rely on the values of flexural strength obtained from three-point bending tests, as advertised by the manufacturer, to predict the performance of polymeric crowns. Although the CTC does not duplicate in vivo loading, it is likely a better representative than flexural tests. It is evident that further investigation is necessary to consolidate and establish the appropriate test methodologies for the determination of dental material strengths to gain acceptance in the scientific community. Additionally, considering the increasing use of PEEK in dentistry, the authors recommend further evaluation through clinical trials as a more suitable approach for comparing the reliability of flexural strength. The present study is limited in that only 10 specimens were used for each material and specimens were generated from discs supplied by the manufacturers; therefore, no quality control was performed. Future research should consider these shortcomings and expand the investigation to include additional measures of strength, including fracture toughness.

## 5. Conclusions

Presently, flexural strengths for three commercial dental polymers obtained from three-point bending were investigated for their correlation with failure loads obtained from crunch-the-crown tests. Based on the findings of this in vitro study, the following conclusions may be drawn:

1. The flexural strengths determined via three-point bending did not correlate with the corresponding failure load of the crowns.
2. The TRI specimens presented higher mean failure load and flexural strength as compared to JUV and PEK, likely due to the presence of woven glass fiber reinforcement (63 wt%).
3. The JUV specimens failed due to deformation only, while the TRI specimens failed due to deformation and cracks. The PEK specimens failed due to fracture.

## Figures and Tables

**Table 1:** The abbreviation, manufacturer, color, class, Young modulus, and Poisson ratio of the test materials.

Test Materials	Abbreviation	Manufacturer	Young Modulus (GPa)	Poisson Ratio
Trilor	TRI	Bioloren S.r.l., Saronno (Varese), Italy	26	0.25
Juvora	JUV	JUVORA Dental, Lancashire, UK	4	0.36
Pekkton	PEK	Cendres + Métaux SA, Biel-Bienne, Switzerland	5	0.38
Epoxy Resin	Epoxy	American Dental Supply, Inc. Allentown, PA, USA	4	0.30

**Table 2:** Classification of structural/mechanical properties according to the specimen geometry and dimensions.

Structural/Mechanical Property	Specimen Geometry	Type of Test	Description	Number of Specimens
Crown failure load (N)	Crown on resin abutment	Crunch-the-crown (CTC)	Maxillary right 1st molar with a thickness of 0.8 mm at the central fossa	10
Three-point flexural strength (MPa)	Bar	Three-point bending (3PB)	Width = 2.0 mm ± 0.2 mm, thickness = 2.0 mm ± 0.2 mm, length = 25.0 mm	10

**Table 3:** Mean values of measured flexural strength (MPa) and crown failure load (N) of TRI, JUV, and PEK with different test configurations.

Test Configuration (Specimen Geometry)		Mean Flexural Strength/Failure Load	SD	Min	Max	Median
3PB test (bar)	TRI	468 MPa ^a,^*	97	378	693	445
	JUV	197 MPa ^b,^*	10	183	216	195
	PEK	192 MPa ^b,^*	15	164	217	191
CTC test (crown)	TRI	7033 N ^a,^*	794	4542	8224	6581
	JUV	5217 N ^b,^*	169	4894	5417	5239
	PEK	3023 N ^c,^*	418	2199	3676	3162

* Different letters within the test (3PB, CTC) indicate that there is a significant difference between the materials. Letter ^a^ means the highest value, and ^c^ means the lowest value, and ^b^ is between a and c. The same order of the letters within the test with different tests confirms the correlation. There was no correlation between the 3PB and CTC tests because they have different orders of materials. TRI = Trilor, JUV = Juvora, PEK = Pekkton, 3PB = three-point bending, CTC = crunch-the-crown.

## Data Availability

The data presented in this study are available on request from the corresponding author.
